# Sequential Effects in Essay Ratings: Evidence of Assimilation Effects Using Cross-Classified Models

**DOI:** 10.3389/fpsyg.2017.00933

**Published:** 2017-06-07

**Authors:** Haiyan Zhao, Björn Andersson, Boliang Guo, Tao Xin

**Affiliations:** ^1^Faculty of Psychology, Beijing Normal UniversityBeijing, China; ^2^Beijing Education Examinations AuthorityBeijing, China; ^3^Collaborative Innovation Center of Assessment toward Basic Education Quality at Beijing Normal UniversityBeijing, China; ^4^School of Medicine, University of NottinghamNottingham, United Kingdom

**Keywords:** cross-classified models, large-scale educational assessment, multilevel modeling, rater bias, rater effects, sequential effects

## Abstract

Writing assessments are an indispensable part of most language competency tests. In our research, we used cross-classified models to study rater effects in the real essay rating process of a large-scale, high-stakes educational examination administered in China in 2011. Generally, four cross-classified models are suggested for investigation of rater effects: (1) the existence of sequential effects, (2) the direction of the sequential effects, and (3) differences in raters by their individual characteristics. We applied these models to the data to account for possible cluster effects caused by the application of multiple rating strategies. The results of our research showed that raters demonstrated sequential effects during the rating process. In contrast to many other studies on rater effects, our study found that raters exhibited assimilation effects. The more experienced, lenient, and qualified raters were less susceptible to assimilation effects. In addition, our research demonstrated the feasibility and appropriateness of using cross-classified models in assessing rater effects for such data structures. This paper also discusses the implications for educators and practitioners who are interested in reducing sequential effects in the rating process, and suggests directions for future research.

## Introduction

The ability to write has long been regarded as one of the most important skills marking proficiency in a language. Therefore, writing assessments are an indispensable part of most language tests. Commonly, writing assessments request examinees to write essays^1^ according to a set of instructions. Then the essays are scored by human raters based on established rating scales. In an ideal, but over-simplified, view of the rating process, raters first internalize a set of stable and uniform standards, and then execute them consistently. Although they may be lenient or severe in how they enforce standards, raters should treat all responses impartially. Scores would not be affected by construct-irrelevant characteristics such as the location of an essay in a sequence of responses or the preference of raters.

However, research has shown that substantial construct-irrelevant variance is introduced into essay scores as a consequence of the rating process alone (Congdon and McQueen, 2000). Even if the rating rubric has been constructed carefully, the reliability and validity of the rating process still depends mainly on the implementation of the rating activities (Overall and Magee, 1992). Because of variations in both the characteristics and status of raters, together with fluctuations between various rating environments, individual raters struggle to remain consistent across multiple rating processes, and different raters may assess the same samples differently. These intra-rater and inter-rater discrepancies can have a negative impact on both the reliability and validity of the resulting scores. Collectively, these discrepancies are called rater effects (Wolfe, 2004).

### Rater Effects and Sequential Effects

Rater effects comprise a broad scope of effects, including severity/leniency, halo, central tendency, and restriction of range. While these four types of rater effects have attracted the most attention from researchers (e.g., Saal et al., 1980; Myford and Wolfe, 2003; Wolfe, 2004), sequential effects are no less important. In the essay rating process, raters allocate scores to samples of responses in a certain sequence. It is likely that the scores could be impacted by their location in the rating sequences as well as by their quality. Sequential effects are said to be present if the score of an essay is affected by previous ratings from the same rater (Jones et al., 2006; Attali, 2011).

Sequential effects may manifest themselves in two ways. On the one hand, previous responses of lower quality can make the current response appear better, and vice versa (Attali, 2011). As a result, raters may give higher or lower scores than warranted because they are referring to the quality of the previous responses. This phenomenon is known as contrast effects (Aiken, 1996). On the other hand, previous low quality responses may cause raters to give lower ratings to current high quality samples, and previous high quality responses may cause raters to give higher ratings to current low quality samples. This phenomenon is known as assimilation effects (Attali, 2011).

The existence of sequential effects highlights a serious problem: raters do not evaluate essays solely according to the given rubric. Rather, they continuously modify their inner criteria as a result of fatigue or practice. Just like all the other well-known rater effects, the harm of sequential effects is serious enough that researchers and practitioners must pay attention to them.

### Sequential Effects in Psychophysics and Essay Ratings

Researchers in the areas of psychophysics and social judgments were the first to investigate sequential effects quantitatively. Most studies in these areas revealed the existence of contrast effects, such as the studies concerning visual perception (Helson, 1964), square sizes (Parducci et al., 1969), attractiveness (Brown et al., 1992; Thornton and Moore, 1993), and fairness (Mellers, 1986), among others. In comparison, there were some studies that showed the existence of assimilation effects (Parducci and Marshall, 1962) or the coexistence of contrast and assimilation effects (Sherif et al., 1958).

Sequential effects were also found in studies of how essays are rated. In the early studies, sequential effects manifested themselves as contrast effects (Hales and Tokar, 1975; Hughes et al., 1980a,b, 1983; Daly and Dickson-Markman, 1982; Hughes and Keeling, 1984; Spear, 1997). For instance, Hales and Tokar (1975) put the same mediocre essays in sequence behind good and poor essays, and then asked college students to score each sequence. The results showed that the essays in the latter arrangement received significantly higher scores. Hughes et al. (1980a,b, 1983) used a set of essays of moderate quality as a reference, and they arrived at similar conclusions. The arrangements in these studies were very similar to those conducted in psychophysics and social judgments, insofar as most of the studies were conducted under experimental conditions where raters were usually inexperienced and untrained, and a limited number of ratings were made. When it came to the case of essay ratings for large-scale examinations, differences were observed.

Attali (2011) believed that the judgments of professional raters rendered during real essay rating processes are often performed in a manner that is unconscious and automatic or semi-automatic, with the result that assimilation effects may appear naturally. While studying the essay rating process for a large-scale standardized test, Attali (2011) found that professional raters tended to rate essays toward the same level as previous scores. Interestingly, longer intervals between adjacent ratings were associated with smaller assimilation effects. Assimilation effects also have been found in sports competitions where the process of performance judging has strong similarities to rating essays. In a study conducted by Damisch et al. (2006) of gymnastics competitions in the 2004 Olympic Games in Athens, the partial correlation between scores of present and previous athletes was 0.31. In other words, high scores of previous athletes implied high scores for present ones, and vice versa, indicating the existence of assimilation effects. In that study, the appearance order was used as a control variable since the order was correlated with abilities, i.e., the order was not random.

### Existing Methods for Exploring Sequential Effects

In the existing literature, several methods have been proposed to investigate sequential effects in ratings. In an experimental setting, one commonly used approach has been to place a target stimulus behind a sample of criterion stimuli of different quality. Then the ratings under the various settings were compared. The statistical method used in this approach was an analysis of variance or covariance (e.g., Hughes et al., 1980a,b, 1983; Daly and Dickson-Markman, 1982; Hughes and Keeling, 1984; Spear, 1997).

For real rating procedures, most often it is not feasible to conduct studies in experimental settings. Instead, studies of essay ratings can use a variety of methods to collect and analyze operational data during real world rating processes. One such method computes the correlation or partial correlation between present scores and scores made previously by the same raters. A significant positive correlation would indicate the existence of assimilation effects, whereas a significant negative correlation would indicate the occurrence of contrast effects (Damisch et al., 2006; Attali, 2011). However, the drawback for use of the correlation method is that it is unable to examine the joint influence of multiple previous scores on the present score. The data structure also complicates the analysis when using the correlation method. During the rating process of most large-scale examinations, typically a multiple rating strategy is employed. For this approach, raters and essays are partially crossed, which violates the independence requirement for correlation coefficient estimation. If the data structure is not taken into account, the resulting standard errors of the parameter estimates will be underestimated, which will lead to inflated type I error rates (Barcikowski, 1981; Scariano and Davenport, 1987; Raudenbush and Bryk, 2002).

Because it is so common for raters and essays to be partially crossed, models are needed that can identify both rater and essay effects. Cross-classified models (Raudenbush, 1993; Rasbash and Goldstein, 1994; Browne et al., 2001) have been proposed to handle such structures. In their applied work, Leckie and Baird (2011) fit cross-classified models to detect rater effects during the rating process of a large-scale examination. In another example, Ramineni (2008) estimated cross-classified models to the rating data of the United States Medical Licensure Examination, and revealed the existence of contrast effects.

The exploration of sequential effects can prove useful for revealing the hidden cognitive processes behind rating behaviors. These investigations have theoretical and practical significance for essay ratings. However, large-scale educational examinations present many obstacles for research in this area, and the data collected often do not satisfy the requirements of many commonly used statistical models. Recognizing the significance of investigating sequential effects in essay ratings and nature of the data structures found in large-scale examinations, our study aimed to explore potential sequential effects in essay ratings by using cross-classified models.

### The Present Study

The goal of this research was to explore sequential effects in essay ratings in large-scale, high-stakes educational examinations. The study was designed to illustrate the feasibility and appropriateness of cross-classified models in assessing rater effects for such data structures. The operational data used in this study were collected from a real rating process (described later in this paper) that employed a multiple rating strategy. The following research questions were addressed:

(1) Were the sequential effects of previous scores on subsequent scores positive or negative?
(2) Did raters differ in the sequential effects they demonstrated?
(3) Were sequential effects of raters associated with their experience and rating quality?

The remainder of this paper is organized as follows. In the next section, we introduce a data structure that occurs commonly in large-scale essay ratings, along with details of the cross-classified models for handling such a structure. Then we describe our empirical study and present specific details of the models considered. Last, we provide a discussion of the results. The paper closes with our conclusions and suggestions for future work.

## Materials and Methods

### Cross-Classified Structures

**FIGURE 1:**
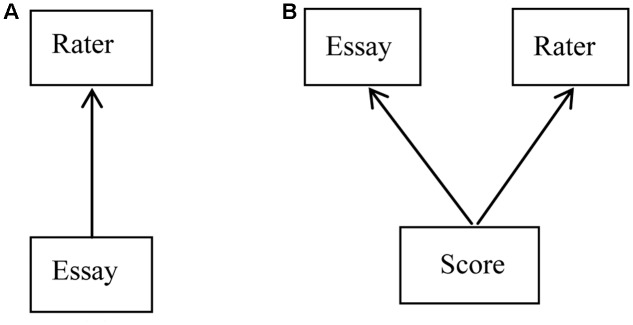
Classification diagrams for **(A)** simple 2-level nested structure and **(B)** cross-classified structure.

Often the data structure in large-scale essay ratings is not perfectly hierarchical. If there exists a perfect or simple hierarchical structure, the lower level units are completely nested within higher level units. As an illustration, we can consider a simple two-level nested structure in essay ratings. Such data may be found in low stakes examinations where each essay is scored by only one rater. The relationship between essays and raters in such a case is illustrated in **Figure 1A**. In **Figure 1**, the units of different levels are represented by boxes, and the classification between units of different levels is represented by arrows from the lowest level units to the classification units.

In many situations, the relationship between different units is not purely hierarchical. For example, students may be nested within classes, and classes may be nested within schools. However, students may also belong to more than one type of unit at a given level of a hierarchy, as when a student belongs sequentially to a particular primary school and secondary school. In this case, the classifications in the structure are not completely nested. Such a structure is known as a cross-classified structure. For essay rating, a cross-classified structure is common. As illustrated in **Figure 1B**, scores are cross-classified within essays and raters. Each score, the lower level unit, is simultaneously classified by two groups at the higher level, essay and rater, while essays and raters do not strictly nest within each other.

### Cross-Classified Models

For data with a cross-classified structure, the application of cross-classified models has been recommended in order to adjust for the data non-independence, improve the quality of estimates of explanatory variable effects, and identify components of variance in the outcomes more accurately (Raudenbush, 1993; Rasbash and Goldstein, 1994; Browne et al., 2001; Fielding and Goldstein, 2005). We proposed the following model for the essay ratings to account for the complex hierarchical structure with random cross-classifications:

$$\begin{array}{c} {score}_{ijk}=(X\beta )+u_{essay\left(j\right)}^{\left(2\right)}+u_{rater\left(k\right)}^{\left(3\right)}+e_{ijk},\\ u_{essay\left(j\right)}^{\left(2\right)}∼N(0,\sigma_{u\left(2\right)}^{\left(2\right)}),u_{rater\left(k\right)}^{\left(3\right)}∼N(0,\sigma_{u\left(3\right)}^{\left(2\right)}),\\ e_{ijk}∼N\left(0,\sigma_{e}^{2}\right) \end{array}\}$$

where *score*_ijk_ represents the *i*-th score for the *j*-th essay given by rater *k. X* is the vector of explanatory variables, and β is the vector of slope parameters. The random part of the model consists of two level-2 error terms, one for the essay $\left(u_{essay(j)}^{\left(2\right)}\right)$ and one for the rater $\left(u_{rater(k)}^{\left(3\right)}\right)$, along with the usual level-1 error term for each score (*e_ijk_*). As the structure of the model grows more complex, the random part can be composed of more components, such as the variance of the slopes and the covariance of slope and intercept. Moreover, the variance in level-1 can have a complex pattern, e.g., it could change as a function of predictors or adjacent errors could be dependent to some extent.

### Methods for Estimation and Model Comparison

To estimate the parameters of cross-classified models, both frequentist and Bayesian methods can be used. Rasbash and Goldstein (1994) proposed a likelihood-based approach that transformed the cross-classified model into a constrained nested model, and then used an iterative generalized least squares algorithm (IGLS) to estimate. Other frequentist approaches included the alternating imputation prediction method (Clayton and Rasbash, 1999), Gauss–Hermite quadrature within penalized quasi-likelihood (PQL) estimation (Pan and Thompson, 2000), and the HGLM framework (Lee and Nelder, 2000). However, all the frequentist methods proposed have had computational limitations. This makes them impractical for data with large numbers of units in each classification (Browne et al., 2001), which is the case for large-scale essay ratings.

However, these limitations can be overcome by using Bayesian methods. Bayesian estimation can be implemented for cross-classified models using the Markov Chain Monte Carlo (MCMC) technique, in which each classification is treated as a random additive term. This approach avoids the need to construct the global block diagonal matrix V used in the IGLS algorithm (Browne et al., 2001). Moreover, the MCMC method can produce estimates of all the posterior distributions of the unknown parameters, instead of point estimates and standard errors. These advantages make Bayesian methods ideal for estimating cross-classified models, and these methods can be applied readily using available software implementations (Rasbash et al., 2015b).

When estimating models with the MCMC algorithm, the deviance information criterion (DIC) is recommended for model comparisons (Spiegelhalter et al., 2002). The DIC is a generalization of Akaike’s information criterion (Akaike, 1974) that can be used to compare both non-nested models and models that have the same response but different structures. With the DIC, a lower value corresponds to a better model fit.

### The English Test

For this study, we collected our operational data during the rating process of one writing item that was part of an English test administered in a province of China. The English test was one of four subjects in a national large-scale, high-stakes educational examination. A student’s total raw score on the four tests as a whole served as the sole criterion for whether the student would be allowed to enter a college or university. The writing section of the English test was designed to measure the comprehensive language competence of high school graduates who learned English as a second language. The writing section was composed of two items. Our study focused on the first item, which was scored on a 20-point scale. This item provided a series of pictures describing an event, based on which students were instructed to write an essay of no less than 60 words with a time limit of 30 min.

The present study was carried out in accordance with the Helsinki Convention and the Norwegian Health Research Act. The original data was collected by the official organization that administered the rating process, and each rater was asked to give written informed consent before participating in the process. The protocol was reviewed and approved by the Ethics Committee of Beijing Normal University.

### The Rating Process

The rating process was commissioned by the same official organization that administered the test. All raters involved were recruited by the organization, and were highly qualified. The eligibility requirements for potential raters included a bachelor’s degree in English, no less than 1 year teaching experience, and district level rating experience. There were 88 raters in the rater group. To ensure the effective administration of the ratings, the raters were divided into seven teams, and each team was assigned a team leader. In allocating raters to teams, the age, gender, and educational districts were taken into account to ensure the homogeneity of the teams.

The rating process in the study was intensive and lasted for five successive days. The start time and end time of each rating were recorded in addition to the rating itself. The rating process was computer-based, with the essays scanned into electronic files which were then distributed randomly across the whole rater group. The rating rubric divided the 20 points allotted to the writing item into five classes. Scores in the range of 16 to 20 points were defined as “high,” and constituted the first (top level) class.

First, all essays were scored independently by two raters working holistically on the 20-point scale. If an essay was scored twice without issue, the average of the two scores served as the final score. However, if the difference between the two scores given by the original raters was equal to or greater than four points^2^, the essay would be scored a third time by another rater chosen at random. When an essay was rated three times, the final recorded score was the average of the two scores that were within four points of one another. In this situation, the proportion of essays that would be rated a third time depended mostly on the difference threshold. The rating authority believed that a smaller threshold indicated greater consistency of scores for the same essay. Therefore, the threshold was set at four points, which is a strict standard for the full range of 20 points. In the present rating process, 17% of the essays were rated a third time. Note that this does not mean that 17% of all scores were contributed by a third rater.

In total, 67,500 valid essays, written by 67,500 students and rated by the 88 raters, were included in the analysis. Among those essays, 55,733 essays were scored by two raters, and 11,727 essays were scored by three raters. Consequently, 146,727 scores were generated.

### The Definition of Models

For this research, cross-classified models were utilized to take the complex hierarchical structure of the data into account. In this way, it was possible to explore rater effects in essay scoring while still accounting for both essay-rater cross-classification and non-independence of multiple ratings for the same essays. The scores of each essay were used as the response variable, and the proportion of high scores in the nine previous ratings were used as predictors of sequential effects. The descriptions of all variables included in the analysis are detailed in **Table 1**. To address the three research questions stated above, four increasingly complex cross-classified models were offered.

**Table 1:** Description of variables included in the cross-classified models.

Name	Description	Level	*N*
*score*	Scores of the present essay item	Score	146,727
*verbal*	Scores of the verbal section	Essay (student)	67,500
*writing*	Scores of the other essay item in the same test	Essay (student)	67,500
*highpro_9*	The proportion of high scores in the nine previous scores	Score	146,727
*experience*	Times of rating similar tasks	Rater	88
*trirate*	The proportions of essays rated by a third rater	Rater	88
*scoremeans*	The means of all scores made by a rater	Rater	88


Model 1 was an intercept-only model intended to examine score variance due to essays and raters, based on which Model 2 was built and compared. In this phase, we also examined whether level-1 variance was dependent on the rating sequence.

Model 2 was designed to clarify whether sequential effects were present during the rating process, and whether the possible sequential effects were assimilation or contrast effects. This model included three predictors: *verbal, writing*, and *highpro_9*. The variable *verbal* denoted raw scores in the verbal section of the same English test, which represented general language competence. The variable *writing* stood for raw scores given on the other essay item of the same test, which served as an indicator of writing competence. The variable *highpro_9* represented the proportion of high scores (i.e., scores in the range of 16 to 20 points) in the nine scores preceding the present score in the rating sequence of the same raters. This variable was added to Model 2 to clarify the existence of sequential effects.

The adoption of *highpro_9* as the predictor for sequential effects was inspired by the research of Attali (2011), in which nine previous scores were correlated with the present one, and the work of Ramineni (2008), in which the proportions of extreme scores were used as predictors. Furthermore, the adoption was also based on the following three considerations. First, the proportions of high scores represented the joint influence of several previous scores, and their use also avoided the potential problem of collinearity when nine individual scores were added simultaneously to the model. Second, the adoption conformed to the nature of the rating process. During the present rating process, all raters were asked to follow three steps before achieving a final score: read an essay, allocate it to one of five classes, and calibrate a final score within the class. During the intensive and exhausting rating process, it was easier for raters to have an overall impression of the several previous scores they gave. Finally, in a high stakes educational examination like the present test, a difference of one or two points within the higher class could decide whether a student would be allowed to enter a college or university. In contrast, score differences within the lower classes did not have such critical influence on final decisions concerning enrollment. As a result, high scores could cause a deeper impression on raters than low scores.

In Model 2, the slopes for all three predictors were set to be fixed. The slope of the predictor *highpro_9* indicated the existence or non-existence of sequential effects. If the credible interval for the slope did not include zero, then the proportion of high scores among the previous nine scores were associated with the present scores. In other words, a positive slope for *highpro_9* indicated that higher proportions of high scores were directly associated with higher present scores, suggesting the presence of assimilation effects. Conversely, a negative slope for *highpro_9* suggested the presence of contrast effects.

In Model 3, the slope of *highpro_9* was allowed to vary across raters in order to explore whether or not there were individual differences with respect to sequential effects. If the credible interval for the resulting variance of slopes did not include zero, that result provided evidence that raters differed in sequential effects expressed by the association between the proportions of high scores and the present scores.

Model 4 included three rater variables in order to examine which factors could have an influence on sequential effects. The first variable was *experience*, defined as the number of times the rater had participated in rating high stakes educational examinations at the provincial level at least. The second variable was *scoremeans*, an indicator of severity, defined as the mean score of all essays scored by a rater. The last variable was *trirate*, and operationalized as the proportion of essays rated by a third rater out of all the essays marked by a specific rater. In addition to considering these variables in the cross-classified models, interaction terms between the rater variables and *highpro_9* were considered.

All models in this study were estimated using the MLwiN (2.34) MCMC procedure at its default setting (Rasbash et al., 2015a). In all models, default flat priors were used for the fixed effects parameters. Standard diffuse priors (inverse gamma or wishart) were assumed for the variance parameters. Both the burn-in length and the sample chain length were set as 50,000. Convergence was monitored and explored for each model by checking information on MCMC trajectory plots, such as the autocorrelation function (ACF), the partial autocorrelation function (PACF), the Raftery–Lewis diagnostic, the Brooks–Draper diagnostic, and the effective sample size measure (ESS) (Rasbash et al., 2015b). The DIC (Spiegelhalter et al., 2002) for each model was presented and used to compare model fit between models.

## Results

### Existence of Sequential Effects

Model 1 included only a constant term in the fixed part. As shown in **Table 2**, significant variance existed between both the raters [$\sigma_{u\left(3\right)}^{2}=0.309$, 95% credible interval (CrI): (0.227, 0.420)] and the essays [$\sigma_{u\left(2\right)}^{2}=9.983$, 95% CrI: (9.779, 10.181)]. Considerable residual variance was also present [$\sigma_{e}^{2}=8.962$, 95% CrI: (8.887, 9.038)]. The between-rater variance was much smaller than the between-essay variance, indicating that the influence of raters on scores was much smaller than the influence of essays themselves. The former accounted for only 1.6% of the total variance, while the latter accounted for 51.9%. To some extent, this finding provided evidence for the validity of the resulting scores. Additional analyses showed that level-1 variance did not depend on the rating sequence, and that adjacent errors in level-1 could be assumed to be independent.

**Table 2:** Parameter estimates (SE) for model 1 and model 2.

	Model 1	Model 2
Fixed		
Constant (β_0_)	8.725 (0.062)	8.596 (0.051)
(*verbal*-gm) (β_1_)		0.077 (0.001)
(*writing*-gm) (β_2_)		0.698 (0.004)
*highpro_9* (β_3_)		1.788 (0.037)
Random		
Between rater variance $\left(\sigma_{u\left(3\right)}^{2}\right)$	0.309 (0.050)	0.229 (0.036)
Between essay variance $\left(\sigma_{u\left(2\right)}^{2}\right)$	9.983 (0.102)	1.744 (0.028)
Residual variance $\left(\sigma_{e}^{2}\right)$	8.962 (0.039)	4.910 (0.021)
DIC	765936.1	668997.4
DIC change (compared with the precious adjacent model)		-96938.4


Model 2 added three fixed covariates: *verbal*, *writing*, and *highpro_9*. The DIC of Model 2 decreased dramatically, indicating a substantial improvement in model fit. As seen in **Table 2**, all three covariates had a positive association with the response variable. The effect predictor *highpro_9* had a positive influence [β_3_ = 1.788, 95% CrI: (1.716, 1.863)]. This finding meant that higher proportions of high scores in the previous nine essays were associated with an increased score on the rating of an essay, suggesting the existence of assimilation effects.

Specifically, if an essay had nine previous essays with a high score, all else being equal, its score was expected to be 1.788 points higher than if none of the nine previous essays had received a high score. On the whole scale of the present essay item, the estimated effect (1.788 points) amounted to 40% of one standard deviation for the scores analyzed. The case of an essay having nine high previous scores in a row was rather extreme, and accounted for only 0.2% of the total cases. The cases with four or more high previous scores accounted for 18.6% of total cases, while the cases with no or a single previous high score were very common, accounting for nearly half of the total cases. Hence, comparing the more common setting of four previous high scores to the case of one previous high score, the score of the target essay was expected to be 1.788 × (0.44-0.11) = 0.590 higher, about 13% of one standard deviation for the present scale.

### Evidence of Individual Differences in Assimilation Effects

**FIGURE 2:**
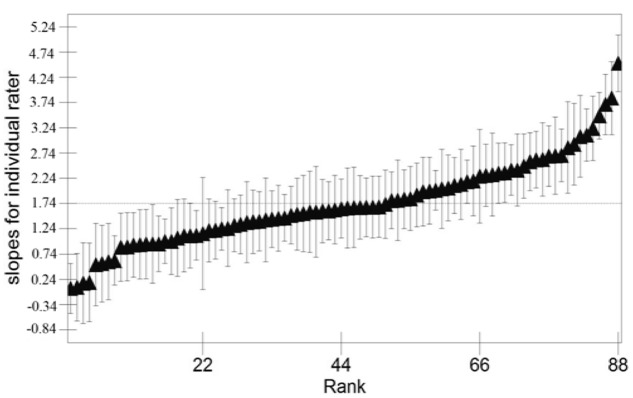
Slopes of *Highpro_9* for individual raters (black triangle) plotted in ascending rank. Each slope is presented with a 95% credible interval (vertical thin line).

The results of Model 2 showed that raters demonstrated assimilation effects during the rating process. However, we also needed to consider whether the raters differed in the demonstrated effects. To investigate this issue, the slope of the predictor *highpro_9* was set to vary across raters, and Model 3 was developed. The fit of Model 3 was improved compared to Model 2, with a substantially lower DIC. The slope of *highpro_9* was still positive [β_3_ = 1.740, 95% CrI: (1.522, 1.949)], and the results from fitting Model 3 indicated that not only were sequential effects present in terms of assimilation effects, but the strength of these effects also varied between the raters [$\sigma_{u(3)\left(3,3\right)}^{2}=0.857$, 95% CrI : (0.608, 1.199)]. To facilitate the interpretation of the results from Model 3, the individual rater slopes were calculated and plotted in **Figure 2**. In the figure, the slopes were ranked and plotted with the corresponding 95% credible interval (vertical thin lines). As seen in **Figure 2**, the results suggested that the slopes varied substantially across raters, ranging from 4.516 [95% CrI (3.954, 5.079)] to 0.061 [95% CrI (-0.433, 0.536)]. Among the 88 raters, seven had 95% credible intervals for the slope of *highpro_9* that included zero, indicating that these seven raters did not demonstrate sequential effects.

### The Influence of Experience and Rating Quality on Sequential Effects

During the rating process, information about the raters was collected. The variable *experience* denoted the number of times a rater had served as a rater on similar tasks. The variable *trirate* denoted the proportion of essays that was rated by a third rater, and the variable *scoremeans* denoted the average score awarded by a rater. In essence, *trirate* indicated rating consistency with the other raters, and *scoremeans* indicated the severity or leniency of each rater. In order to explore whether rater characteristics had an effect on the estimated sequential effects, the main effect term of the three rater variables was included in Model 4, along with the interaction terms between the rater variables and *highpro_9*. All slopes of the newly added terms were set to be fixed. The results are given in **Table 3**. Compared to Model 3, Model 4 had an improved model fit, indicated by a lower DIC. Model 4 was the best-fitting model among all those considered. Compared to Model 3, the between-rater variance was reduced by 80.2%, and the variance of the *highpro_9* slopes decreased by 48.3%. Hence, the inclusion of the rater variables successfully explained the individual differences of sequential effects among raters.

**Table 3:** Parameter estimates (SE) for model 3 and model 4.

	Model 3	Model 4
Fixed		
Constant (β_0_)	8.618 (0.064)	8.661 (0.031)
(*verbal*-gm) (β_1_)	0.077 (0.001)	0.077 (0.001)
(*writing*-gm) (β_2_)	0.698 (0.003)	0.698 (0.003)
*highpro_9* (β_3_)	1.740 (0.109)	1.692 (0.082)
(*experience*-gm) (β_4_)		0.017 (0.017)
*highpro_9 ×* (*experience*-gm) (β_5_)		-0.117 (0.047)
(*scoremeans*-gm) (β_6_)		0.773 (0.054)
*highpro_9 ×* (*scoremeans*-gm) (β_7_)		-0.355 (0.145)
(*trirate*-gm) (β_8_)		-5.358 (0.906)
*highpro_9 ×* (*trirate*-gm) (β_9_)		16.235 (2.426)
Random		
Between-rater variance $\left(\sigma_{u(3)\left(0,0\right)}^{2}\right)$	0.349 (0.055)	0.069 (0.012)
Variance of *highpro_9* slope $\left(\sigma_{u(3)\left(3,3\right)}^{2}\right)$	0.857 (0.151)	0.443 (0.092)
Covariance of *intercept* and *highpro_9*slope $\left(\sigma_{u(3)\left(3,0\right)}^{2}\right)$	-0.362 (0.077)	-0.095 (0.027)
Between-essay variance $\left(\sigma_{u(2)\left(2\right)}^{2}\right)$	1.739 (0.028)	1.738 (0.028)
Residual variance $\left(\sigma_{e}^{2}\right)$	4.886 (0.021)	4.886 (0.021)
DIC	668338.7	668324.3
DIC change (compared with the precious adjacent model)	-658.7	-14.4


As shown in **Table 3**, the slope of *highpro_9* was still positive [β_3_ = 1.692, 95% CrI: (1.527, 1.849)] and still varied among the raters [$\sigma_{u(3)\left(3,3\right)}^{2}=0.443$, 95% CrI : (0.291, 0.646)] when the new variables were included in the model. Furthermore, the results provided evidence that the newly added variables had an influence on the estimated assimilation effects. First, although *experience* had no significant main effect on the response variable [β_4_ = 0.017, 95% CrI: (-0.017, 0.050)], it could moderate the influence of *highpro_9*, i.e., a unit increase in experience would reduce the effect of *highpro_9* by 0.117 [β_5_ = -0.117, 95% CrI: (-0.209, -0.028)]. These results suggested that even if raters with various levels of experience did not differ in terms of severity or leniency, the raters did differ in their inclination to give scores that were influenced by previous scores they had given. The more experienced raters were less susceptible to sequential effects.

Second, *scoremeans* had a positive effect on the response variable [β_6_ = 0.773, 95% CrI: (0.665, 0.879)], indicating that the more lenient the raters were, the higher the scores they gave, which was consistent with the operationalized definition of the variable. The results also implied that *scoremeans* had a negative impact on assimilation effects since an increase in *scoremeans* by one point was associated with a decrease in the slope of *highpro_9* by 0.355 [95% CrI: (-0.640, -0.062)]. The variable *scoremeans* had a range of 7.71 to 10.22, which meant that the difference between the most lenient and most severe raters would have a difference in the slope of *highpro_9* of 0.89, nearly half of the effect estimated for the rater group as a whole. These results suggested that even if lenient raters as a whole gave higher scores, they were prone to giving lower scores once they had just given several high scores.

Finally, the variable *trirate* was negatively associated with scores [β_8_ = -5.358, 95% CrI: (-7.144, -3.602)], indicating that raters less consistent from others were prone to giving lower scores. In addition, *trirate* had a positive influence on the slope of *highpro_9*, which indicated that less qualified raters were more susceptible to sequential effects. When *trirate* increased by 0.01 (*trirate* was expressed as a percentage and ranged between 0.11 and 0.26, with a mean of 0.17), the slope of *highpro_9* increased by 0.162 [β_3_ = 16.235, 95% CrI: (11.441, 20.878)]. More specifically, there would be a difference of 2.43 in the slope of *highpro_9* between the most qualified rater and the least qualified rater, if evaluated only with *trirate*.

## Discussion

The results of this study strongly suggested that cross-classified models have an advantage over other methods for investigating rater effects in real essay rating processes for large-scale, high-stakes educational examinations. Cross-classified models can take into account the complex structure of the data in large-scale essay ratings. Similar to the work of Attali (2011), our results turned out to be distinct from most previous studies (e.g., Hales and Tokar, 1975; Hughes et al., 1980a,b, 1983; Daly and Dickson-Markman, 1982; Hughes and Keeling, 1984; Spear, 1997). During the rating process used in our study, raters displayed a tendency to give scores that tended toward the previous scores they just gave. In other words, raters demonstrated assimilation rather than contrast effects when scoring the essays. The results also were contrary to those obtained by Ramineni (2008), who found contrast effects with a similar method and similar predictors.

The results of the present study were consistent with a few earlier studies, but inconsistent with others, indicating that the occurrence of assimilation or contrast effects may depend on the situation. Several existing studies suggested that assimilation effects tended to take place in situations when perceived similarities existed between target and reference stimuli (Mussweiler, 2003), when judges were confident and certain about their judgments (Pelham and Wachsmuth, 1995), and when judges lacked motivation or cognitive resources to compare targets with references (Martin et al., 1990). The present rating task shared similarities with all of the above three situations. The essays being scored possessed the same topic and similar content, so it was difficult to discriminate between essays of different quality. All raters had direct experience and were skilled in scoring essays. Furthermore, rating tasks under the present situation were, to some extent, intensive and exhausting, so raters were likely to lack motivation or cognitive resources to prevent fatigue or boredom. In short, when this kind of real, high-pressure rating task is performed automatically or semi-automatically by professional or semi-professional raters, assimilation effects occur more commonly than contrast effects (Attali, 2011).

Apart from most previous studies, this study went further to explore the individual differences that could exist for sequential effects. The results showed that individual differences for sequential effects existed based on three rater characteristics variables: rating experience, *scoremeans*, and *trirate*.

• Raters with different levels of experience differed from each other. Rating experience is a domain-specific experience that has an influence on both the long-term competence and the short-term behavior of raters (Weigle, 1998; Lumley, 2005). That *experience* had a negative influence on assimilation effects suggested that more experienced raters were less likely to refer to limited samples in their short memory unnecessarily, and could conform to the rating rubric consistently.
• The negative influence of *scoremeans* indicated that lenient raters were less susceptible to assimilation effects, which was not in line with the observation that lenient raters usually gave higher scores. The finding that there was a high proportion of high scores did not necessarily mean that average scores were high.
• The variable *trirate* was included in the model to represent the degree of consistency of the present rater with all the other raters as a group. This variable’s positive influence on assimilation effects revealed the relationship between assimilation effects and rating quality. Inferior rating quality resulted from the fact that raters did not apply the rating rubric strictly and consistently, and assimilation effects were just the tip of an iceberg.

Differed in the sequential effects they demonstrated, the more experienced, lenient, and qualified raters were less susceptible to assimilation effects. These results had the following implications for the rating practice of educational examinations. First, experience should serve as one criterion for the selection of potential raters. Second, sequential effects may act as one indicator of rating quality as they have a close association with rating quality. Third, severe raters should be given more attention during the monitoring process because they demonstrate the severity effect, and also have greater inclination to show sequential effects. Fourth, because sequential effects resulted from the tension and anxiety of the rating process to some extent, changing the raters’ working pace might help to reduce the effects. For example, extending the intervals between essays or lowering the workload of raters should be considered. Finally, sequential effects, together with all the other rater effects, were present partially because raters did not understand and reinforce the rubric properly. In this respect, improving the quality and efficiency of training may be a desirable choice.

Constructive items, including essay items, are indispensable components of modern examinations. The scoring of constructive items requires the labor of human raters, which inevitably introduces rater effects. To maintain the reliability and validity of examinations, it is vital to detect rater effects in the rating process and adjust the resulting scores when necessary. Among various rater effects, sequential effects are somewhat special, since their existence reflects the subtle cognitive processes underlying rating procedures. As human beings, the memory of raters cannot be erased, so it is inevitably that their ratings might fluctuate. Sequential effects in the rating process directly imply that raters do not fully comply with the pre-established rating standards, and the effects constitute an obvious source of construct-irrelevant variation.

Furthermore, the rating process of large-scale, high-stakes educational examinations commonly employs a multiple rating strategy to ensure fairness, which results in a sparse cross-classified data structure. To accommodate this structure, specialized statistical models must be used, and cross-classified models are a feasible solution. In this paper, in addition to detecting sequential effects in essay ratings, we have also sought to demonstrate to researchers and practitioners specializing in essay rating and other subjectively evaluated performance tasks that cross-classified models are appropriate and feasible to apply when the data have this type of structure. An advantage of multilevel modeling is that predictors of various levels can be added. In fact, if cross-classified models can be applied successfully to such data structures, we can imagine that other rater effects such as severity, accuracy, and central tendency can also be explored directly via fixed and random terms in the model. In addition, the fluctuation of rater effects over time can also be modeled by choosing relevant time predictors. Such extensions are of great interest for future research.

## Conclusion And Future Direction

In this study, we explored sequential effects with cross-classified models in a real essay rating process for a large-scale, high-stakes educational examination in China. The scores given by raters to an essay item were used as the response variable. The proportion of high scores among the nine previous scores made by the same rater was used as the predictor of sequential effects. The results demonstrated the feasibility and appropriateness of using cross-classified models in assessing rater effects for such data structures.

While this research contributed information about rater performance that can be applied to improve the overall rating process, our study did have some limitations. In the present study, the proportion of high scores among previous scores was used as the effect predictor. Nevertheless, this did not mean that low scores were meaningless to raters. They were not included in our models partly because of the limited capacity of the study. Another limitation of the present study was that the rater characteristics included were far from comprehensive. Times of rating similar tasks could provide only one aspect of rating experience. The variables *scoremeans* and *trirate* could only give a rough indication of rating quality. Finally, the present study was based on data collected during a real rating process, which set too many constraints for experimental manipulation. Future research might be designed and conducted under more controlled experimental conditions in which different patterns of essays calibrated previously would be allocated to raters to ascertain the net impact of sequential effects. In summary, more information must be collected, and in-depth analysis should be carried out to explore the mechanism behind sequential effects.

## Author Contributions

All authors listed, have made substantial, direct and intellectual contribution to the work, and approved it for publication.

## Conflict of Interest Statement

The authors declare that the research was conducted in the absence of any commercial or financial relationships that could be construed as a potential conflict of interest.

## Footnotes

1 In the present study, the term ‘essays’ denotes the responses of examinees to a writing assessment task or a writing item.

2 The difference threshold was set according to official guidelines issued by the National Educational Examination Authority of China. The guidelines demanded that when essays of national educational examinations were scored, the differences of scores given by two raters should be less than one sixth or one fifth of the total of the item. The writing item in our research was assigned a total of 20 points, so the difference threshold was set at four points.
